# Operative treatment of multiple costochondral dislocations in a patient with severe rib fractures and a flail chest following trauma

**DOI:** 10.1136/bcr-2020-239511

**Published:** 2021-03-02

**Authors:** Jonne T H Prins, Mathieu M E Wijffels

**Affiliations:** Trauma Research Unit, Department of Surgery, Erasmus MC, University Medical Center Rotterdam, Rotterdam, Netherlands

**Keywords:** surgery, trauma

## Abstract

A 73-year-old male patient underwent operative treatment for dislocation of multiple costochondral junctions alongside multiple bony rib fractures and a flail chest following high-energy trauma. During the operative fixation of the flail chest, the costochondral lesions were surgically stabilised with plates and screws, which were fixated on the osseous anterior rib, sternum or the rib cartilage. The patient experienced no pulmonary complications during the primary admission. At 7 months after the trauma, the chest CT scan showed full consolidation of all fixated rib fractures, including the costochondral lesions, without hardware dislocation or complications. The patient did not require any pain medication and had no pain during daily activities, at rest or at night. Although being a biomechanically demanding region, which is often not defined in current rib fracture classification, operative treatment of costochondral lesions is feasible with outcome similar to the treatment of bony rib fractures.

## Background

A recent consensus statement of the Chest Wall Injury Society advocated the addition of a costal cartilage sector to the existing anterior, lateral and dorsal sectors to record rib fractures in this anatomic region.^1^ The costal cartilage sector is a biomechanically demanding region due to the symmetrical and asymmetrical tractive, compressive and shearing forces, which can result in sternocostal dislocation and has previously been described after pectus excavatum repair.^2^ Whereas Schulz-Drost has presented satisfactory results after surgical stabilisation of sternocostal dislocation, this operative treatment has, to the best of our knowledge, not been described in the literature for patients with traumatic sternocostal or costochondral dislocation.

This case report presents the surgical stabilisation of multiple dislocated costochondral junctions in a patient with bilateral multiple severe rib fractures after trauma. The patient gave informed consent for submission of this data for publication.

## Case presentation

A 73-year-old man presented at the emergency department after blunt chest and abdominal trauma. He was hit and run over by a minivan. During the prehospital phase, the patient was conscious, haemodynamically stable and had a SpO_2_ of 94% with 15 L on a non-rebreather mask. On arrival at the hospital, the patient had thoracic bony crepitation and a paradoxical breathing pattern on the right side.

## Investigations

CT of the head and cervical spine showed no intracranial abnormalities, but showed various facial fractures. The chest and abdominal CT showed a sternal fracture, multiple simple rib fractures, ribs 2–9, including dislocation of three costochondral junctions, and a scapular fracture on the right side as well as multiple rib fractures, ribs 2–12, with a flail chest on the left side. In addition, a bilateral pneumothorax with right-sided haemothorax and an unstable pelvic ring and sacrum fracture were present. The patient remained haemodynamically stable.

## Differential diagnosis

**Figure 1:**
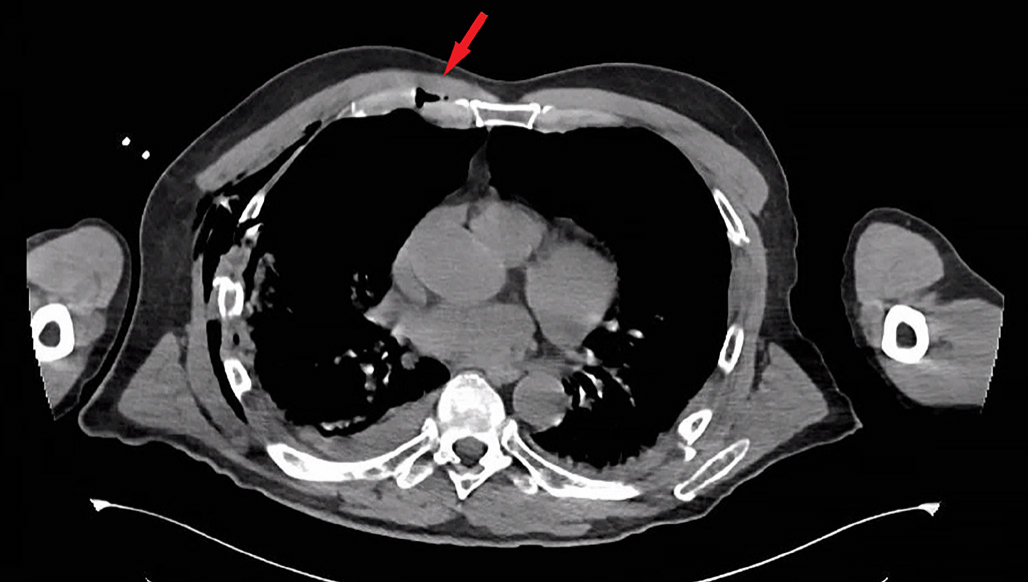
CT scan displaying the costochondral dislocation of the right third rib (arrow).

**Figure 2:**
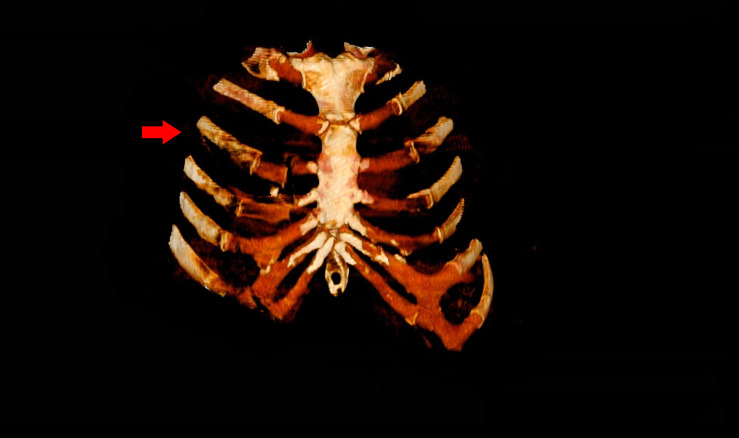
Three-dimensional reconstruction of the chest CT scan showing the dislocated costochondral fractures, starting at the right third rib (arrow).

Multiple costochondral dislocations were visualised on the axial view of the chest CT. At the cartilage section of ribs 3–5, dislocation and air was clearly visible (figure 1). With the intact cortices of the anterior rib and sternal body as well as the three-dimensional reconstruction of the chest CT, the diagnosis of costochondral dislocation was substantiated from the diagnoses of anterior rib fracture and sternal fracture (figure 2).

## Treatment

**Figure 3:**
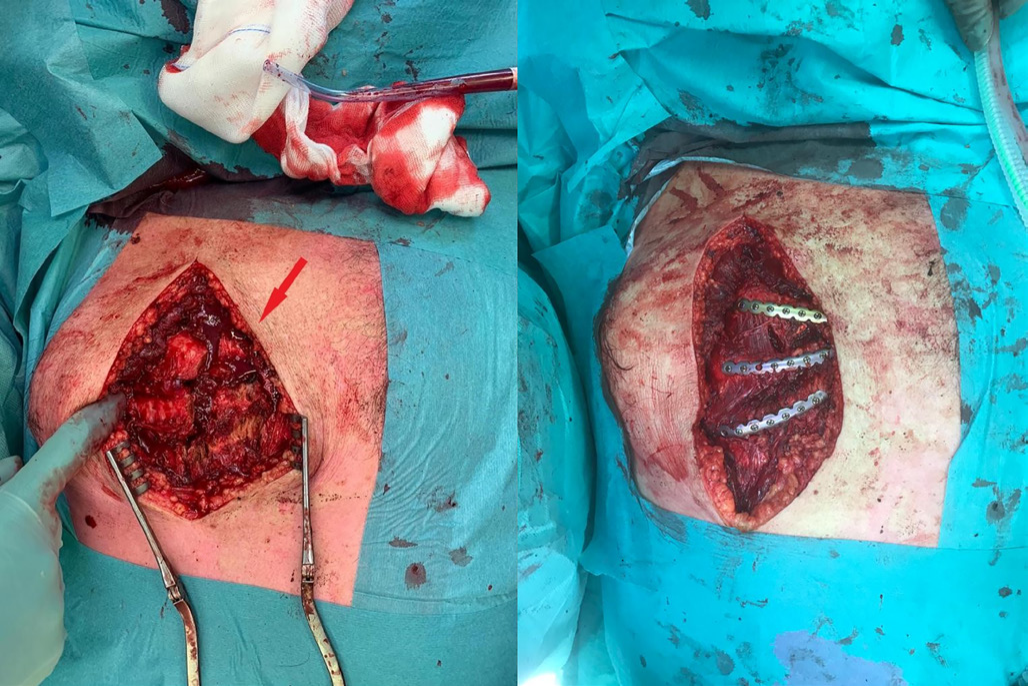
Intraoperative image of the costochondral fractures of right ribs 3–5 (arrow; left) which were fixated with plates and screws (right).

The patient was admitted at the Intensive Care Unit (ICU) and on the same day, the pelvic and sacrum fractures were fixated. The left-sided and right-sided rib fractures were fixated on day 2 after trauma with a transverse muscle-sparing approach, using the MatrixRIB Fixation System (DePuy Synthes, West Chester, Pennsylvania, USA). During the procedure on the right side, the dislocation of the costochrondral junctions of the right ribs 3–5 was visualised (figure 3). To stabilise this costochondral lesion, the patient was placed from a lateral to a supine position and an additional longitudinal parasternal incision was made. Three unstable costochondral junctions were fixated after reposition, spanning the defect from the osseous anterior rib to sternum (rib 3), rib cartilage to sternum (rib 4), and osseous anterior rib to rib cartilage (rib 5), using plates and screws (figure 3).

## Outcome and follow-up

At day 7 after trauma, the patient was extubated and re-intubated on the same day because of pulmonary insufficiency due to atelectasis as a result of a paralytic ileus. He developed no pulmonary or surgical thoracic complications. Eleven days after the chest wall fixation, a tracheostomy was performed to enable weaning from the ventilator, which was successful at 6 days after this procedure. One month after the trauma, the patient was discharged from the ICU after which the clinical course was complicated by a perirectal abscess following the pelvic and sacrum fixation. There were no pulmonary or thoracic complications and the patient was discharged to a rehabilitation centre at 6 weeks after trauma.

**Figure 4:**
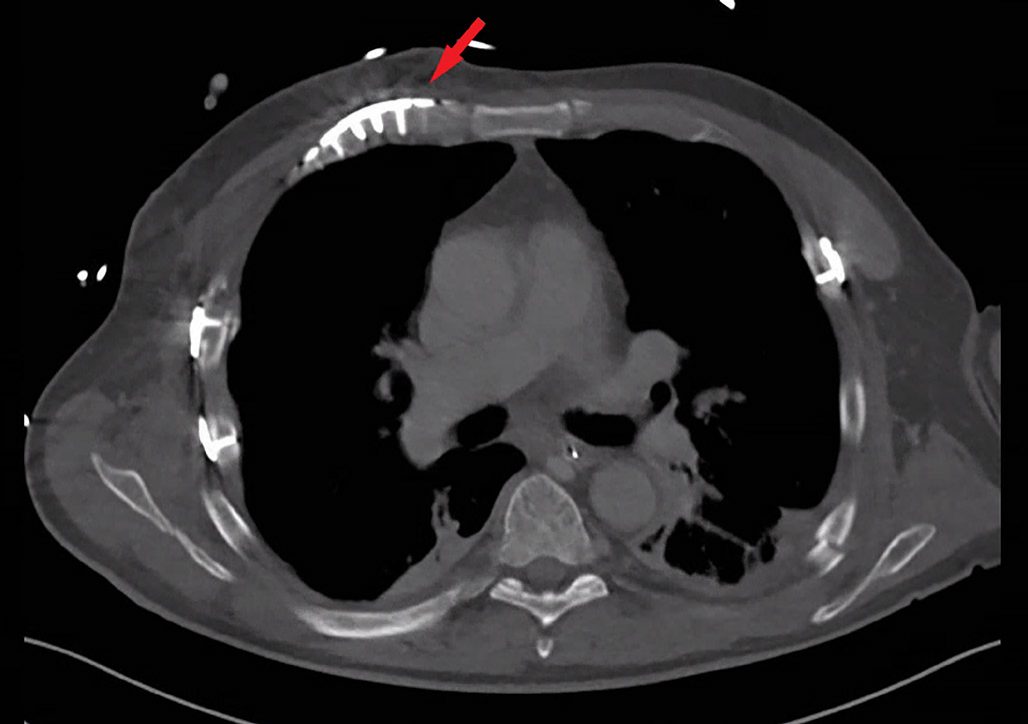
CT scan showing the fixated costochondral defect of the right third rib at 7 months after trauma (arrow).

At 7 months after trauma, the patient was seen at the outpatient clinic. The patient reported no dyspnoea, chest tightness or chest wall numbness. He experienced no chest pain at night, at rest or during daily activities, and did not require any pain medication. The patient described his health as good, with slight problems during self-care and usual activities (eg, tying shoes). The chest CT at final follow-up showed full consolidation of all rib fractures, including the costochondral lesions, without hardware dislocation or complications (figure 4).

## Discussion

This article reports the first case of successful operative management of costochrondral dislocation of multiple rib fractures after a high-energy trauma. Costal cartilage injuries, such as fractures and costochondral dislocation or separation have been described little in the current literature. The prevalence of costochondral fractures on CT after blunt chest trauma is 20%–42% and is more often present in patients with bilateral multiple consecutive rib fractures than those without bilateral rib fractures.^3 4^ This patient had multiple costochondral dislocations concomitant to bilateral anterior and lateral rib fractures, with an additional sternal fracture, bilateral pneumothorax, right haemothorax and cor contusion. Hepatic and aortic injuries, more common in patients with costal cartilage fractures, were not present.^4^

Non-operative management of these traumatic injuries has been addressed in case reports or small case series. Most of these injuries are seen in the young male population and a result of blunt chest trauma, often during contact sports.^5–8^ The five patients described in these four reports were treated non-operatively with good recovery in four. However, one patient reported occasional ‘clicking’ at the injury site 1 year post trauma.^7^ Due to the lack of blood vessels and perichondrium, cartilage is known to have poor healing and union rates after injury.^9^ In animal studies, it has been shown more specifically that traumatic dissection of costal cartilage results in non-united fracture fragments.^10^ Due to beneficial effects over non-operative treatment, interest in and application of surgical stabilisation of rib fractures (SSRF) has risen significantly over the last decade.^11^ With the recognition of a costal cartilage section in rib fracture localisation, the possible favourable effect of SSRF in this type of injury can be studied.^1^ While reporting successful surgical management of costal cartilage fractures, one case report has stressed the technical difficulty of this procedure as the location of this injury often makes the advised bilateral attachment of hardware to bone impracticable.^12^ Another case series of surgically treated patients for sternocostal dislocation after pectus excavatum repair showed no hardware complications and good patient satisfaction at 1 year after treatment.^2^ In this patient, hardware was fixated with screws on the anterior osseous rib surface, costal cartilage and the sternum, offering sufficient compression and grip, resulting in union of all fractures and no material dislocation or complications at 7 months after trauma.

Multiple rib fractures are known to cause long-term pain, morbidity and a significant functional reduction with a low percentage of patients returning to full-time work.^13–15^ With poor healing characteristics and concomitant injuries due to their localisation, costal cartilage fractures or dislocation can be more painful and result in a longer duration of experiencing significant pain than regular costal fractures.^3^ At 7 months after trauma, this patient did experience thoracic pain at night but did not experience any chest tightness, dyspnoea or thoracic pain during daily activities or at rest. In addition, the patient did not use any pain medication and described his health as good. Thus, the outcome of this patient is comparable with outcome after bony rib fractures.

In conclusion, severe chest trauma, resulting in traumatic costochondral dislocation, signifies an interesting entity. Surgical stabilisation of these fractures is technically demanding but was in this case successful. The currently available implants seem to be feasible for these injuries. Further studies, prospective and comparative with non-operative treatment, including larger patient populations focusing on short-term and long-term outcome, are required to show a possible benefit of adding surgical stabilisation to the armamentarium for this type of injury.

**Patient’s perspective**

During the last follow-up moment, we asked the patient to describe his experience of the entire clinical process up to that day. He described this as follows:

Of the first few days or weeks after the trauma, I do not remember much. I was very glad that I was still alive. While my wife and I sometimes joke about how I am some sort of a robot now with all the plates and screws in my body, I am very pleased how my rib fractures healed. Currently, and actually already quickly after hospital discharge, I notice little to no pain of my fractured ribs. For my rib fractures, I did not require pain medication, but since the accident, my knees and hip hurt most. I am on a waiting list to be operated on and get two new knees, but possibly due to the COVID-19 outbreak, this may take a long time. My daily limitations are because of my bad knees and hip. I am very satisfied with the rib fracture treatment and the end result.

**Learning points**

- Costochondral dislocations or fractures may occur following high-energy trauma.
- Costochondral injuries can be delineated and evaluated on chest CT scans or its three-dimensional reconstruction.
- The currently available implants for rib fixation seem feasible for surgically stabilising these injuries with good outcome, similar to that of the treatment of bony rib fractures.
